# Use of verbal de-escalation in reducing need for mechanical restraint in patients with psychotic disorders during non-voluntary transfers from home to the psychiatric emergency department

**DOI:** 10.1192/j.eurpsy.2022.1509

**Published:** 2022-09-01

**Authors:** E. Miranda Ruiz, A. Gonzalez, P. Samos, M. Bellsola, A. Sabate, J. Leon, M.A. Jerónimo, V. Pérez-Solà, L.M. Martin, D. Corcoles

**Affiliations:** 1 CST, Psychiatry, Barcelona, Spain; 2 Hospital del Mar, Psychiatry, Barcelona, Spain; 3 Parc de Salut Mar, Institut De Neuropsiquiatria I Addiccions, Barcelona, Spain

**Keywords:** Emergency psychiatry, Mechanical retraint, Verbal de-escalation, transfers

## Abstract

**Introduction:**

Little is known about the need for mechanical restraint during non-voluntary transfers from patient’s homes to the psychiatric emergency department in patients diagnosed with Paranoid Schizophrenia. Although there is no evidence of its efficacy, one of the main tools used for the reduction of mechanical restraints is verbal de-escalation training.

**Objectives:**

The aim is to describe which symptoms predispose to mechanical restrain in patients with Paranoid Schizophrenia transferred in a non-voluntary manner from home to the psychiatric emergency department, and the effect on reducing mechanical restraints after receiving verbal de-escalation training.

**Methods:**

All patients with Paranoid Schizophrenia who, after being visited by a home psychiatry team, have required non-voluntary transfer from their homes to the psychiatric emergency department were selected (N = 442).

**Results:**

Young age, being male, having a poor adherence to treatment, higher scores for de following variables; Excitement, Grandiosity, Suspiciousness, Hostility, Abstract thinking, Motor tension, Uncooperativeness, Poor attention, Lack of insight and Poor impulse control as well as lower scores in motor retardation on the PANSS, are related to a higher frequency of mechanical restrain (P<0,005). Before the verbal de-escalation training, 43.9% of the transferred patients required mechanical restraint, after the training, the need for restraints was reduced to 25.5% (P<0.001).

**Conclusions:**

Training in verbal de-escalation has allowed an important reduction in mechanical restraints in patients with schizophrenia who have required non-voluntary transfers from home to the psychiatric emergency department.

**Disclosure:**

No significant relationships.

